# Group 2 Innate Lymphoid Cells Participate in Renal Fibrosis in Diabetic Kidney Disease Partly via TGF-*β*1 Signal Pathway

**DOI:** 10.1155/2019/8512028

**Published:** 2019-07-03

**Authors:** Cuiping Liu, Ludan Qin, Jingya Ding, Luping Zhou, Chenlin Gao, Ting Zhang, Man Guo, Wei Huang, Zongzhe Jiang, Yang Long, Yong Xu

**Affiliations:** ^1^Luzhou Key Laboratory of Cardiovascular and Metabolic Diseases, Affiliated Hospital of Southwest Medical University, Luzhou, Sichuan, China; ^2^Department of Endocrinology, Zigong Fourth People's Hospital, Zigong, Sichuan, China; ^3^State Key Laboratory of Quality Research in Chinese Medicine, Faculty of Chinese Medicine, Macau University of Science and Technology, Avenida Wai Long, Taipa, Macau

## Abstract

**Aim:**

To explore the role of group 2 innate lymphoid cells (ILC2s) in the pathogenesis of renal fibrosis in diabetic kidney disease (DKD).

**Methods:**

The proportion of ILC2s and the levels of Th2 cytokines (IL-4, IL-5, and IL-13) in the peripheral blood of normal control subjects (NC) or patients with type 2 diabetes mellitus (DM), early diabetic kidney disease (DKD1), or late diabetic kidney disease (DKD2) were analyzed by flow cytometry and ELISA. The expression of transforming growth factor-*β*1 (TGF-*β*1), fibronectin (FN), collagen1, IL-4R*α*, and IL-13R*α*1 in renal tubular epithelial cells (HK-2) induced by IL-4, IL-13, or high glucose was analyzed by ELISA or qPCR.

**Results:**

The proportion of ILC2s and the levels of IL-4, IL-5, and IL-13 were significantly increased in DKD patients and were positively correlated with the severity of DKD (*P* < 0.05). The expression of TGF-*β*1, FN, and collagen1 was significantly upregulated in HK-2 cells induced by IL-4 or IL-13 (*P* < 0.05). Moreover, the IL-4R*α* and IL-13R*α*1 mRNA in HK2 cells were increased followed by high glucose alone or combined with IL-4 or IL-13, but the differences were not statistically significant (*P* > 0.05). However, compared with high-glucose stimulation alone, the expression of TGF-*β*1, FN, and collagen1 was significantly increased in HK-2 cells induced by high glucose combined with IL-4 or IL-13 (*P* < 0.05).

**Conclusions:**

ILC2s may participate in renal fibrosis in DKD partly via TGF-*β*1 signal pathway.

## 1. Introduction

Diabetic kidney disease (DKD) is the most common cause of end-stage renal disease in the world [1, 2]. Increasing evidences have shown that inflammation and fibrosis are mutually reinforcing and are part of the detrimental process leading to the development and progression of DKD [3, 4]. TGF-*β*/Smad signaling pathway is an important pathway regulating renal fibrosis, and TGF-*β*1 as the most effective profibrotic cytokine can promote the expression of fibrosis-related genes, such as collagen1 and FN [5, 6]. Most importantly, recent studies indicated that immune system activation and imbalance of immune homeostasis play an important role in the development and progression of DKD [7–9]. As the innate mediators of type 2 immunity, ILC2s have been focused extensively [10].

ILC2s are a class of innate, non-B/non-T lymphocytes which derived from a common lymphoid progenitor [11]. ILC2s can act as a bridge between inherent and adaptive immunity [11]. They are also the first class of cells to activate type 2 immunity and play an important role in Th1/Th2 balance [12]. Th1/Th2 imbalance would induce disease; researchers have found that patients with diabetic retinopathy and DKD have Th1/Th2 imbalance [13, 14]. ILC2s not only provide protective immunity toward helminth infection but also are responsible for allergic inflammation, tissue repair, and metabolic homeostasis and mediate multiple types of tissue fibrosis such as intestinal fibrosis, hepatic fibrosis, and pulmonary fibrosis [15–17]. After being stimulated by IL-25, IL-33, and TSLP, ILC2s can secrete Th2 cytokines, such as IL-4, IL-5, IL-9, IL-13, and amphiregulin, even ILC2s are considered the main source of the Th2 cytokines [15, 18]. Moreover, IL-4 can participate in inflammation and fibrosis by regulating cell growth, differentiation, and production of cytokines [19]. And IL-13 exhibits different biological functions in different situations. Apart from its anti-inflammatory role, IL-13 is a strong fibrotic mediator. Many studies have shown that IL-13 can mediate fibrosis of various tissues and organs, such as the skin and liver [18, 20, 21]. So, ILC2s play a key role in regulating fibrosis by secreting Th2 cytokines, including IL-4 and IL-13 [22, 23].

However, the role of ILC2s and Th2 cytokines in kidney disease is controversial. Some studies have demonstrated that ILC2s and Th2 cytokines produced by ILC2s can participate in the protection and repairment of kidney tissue under the stimulation of IL-33 [23, 24]. But another study has proved that ILC2s can produce a large number of Th2 cytokines to mediate the type 2 immunity and subsequently lead to renal tissue damage after being activated by IL-33 [22]. It has been reported that the proportion of ILC2s increased in patients with type 2 diabetic nephropathy on maintenance hemodialysis, compared with T2DM and healthy volunteers [25]. The role of ILC2s in the pathogenesis of renal fibrosis in DKD is still unclear. Therefore, we did this study to explore the role of ILC2s and the Th2 cytokines (IL-4, IL-13) in the pathogenesis of DKD.

## 2. Materials and Methods

### 2.1. Study Participants

With the hospital ethics committee and the patients' consent, thirty type 2 diabetes patients (T2DM) without DKD and thirty patients with DKD who were hospitalized in the Affiliated Hospital of Southwest Medical University from August 2017 to January 2018 were enrolled. Thirty healthy volunteers in the physical examination center were enrolled at the same time. The inclusion criteria were as follows: (1) the subjects were 40-80 years old, (2) patients were diagnosed as T2DM in accordance with the 1999 WHO diabetes expert committee, and (3) patients were diagnosed as DKD according to the criteria provided by expert consensus on the prevention and control of DKD in 2014 [25]. Patients with DKD were grouped according to disease stage with microalbuminuria (≤300 mg/g) defined as early DKD and macroalbuminuria (>300 mg/g) and periods of renal failure defined as late DKD. The exclusion criteria were as follows: (1) nontype 2 diabetes, (2) pregnancy or lactation, (3) acute/chronic heart failure, acute/chronic respiratory failure, all types of viral hepatitis, cirrhosis, and other serious diseases, (4) obvious infectious disease, (5) presence of malignant tumor(s), (6) use of immunomodulatory drugs or immunosuppressive agents for >6 months, (7) autoimmune or rheumatic diseases, or (8) uncooperative. The Chinese Clinical Trial registration number is ChiCTR 1800014519.

### 2.2. Blood Samples

Peripheral blood samples were collected and divided into two sets. One set was centrifuged and frozen at -80°C for future use; another one was utilized for flow cytometric analysis immediately.

### 2.3. Flow Cytometry

ILC2s were defined as Lin^−^CD45^+^CD127^+^CD294^+^ cells. PBMCs (Peripheral Blood Mononuclear Cells) were isolated as described as follows. Heparinized peripheral blood was diluted with 1 : 1 phosphate-buffered saline (PBS) and layered over an equal volume of ficoll as per the manufacturer's instructions. After centrifuged, mononuclear cells were collected from the interface between the ficoll and plasma. The collected PBMCs were washed three times with PBS and resuspended in 200 *μ*L PBS before further manipulation. Obtained PBMCs were enumerated. PBS was added to adjust the concentration of the suspension to 5 × 10^3^/*μ*L. 200 *μ*L aliquot of the obtained PBMCs was incubated with mouse anti-human CD127 antibody (5 *μ*L), mouse anti-human CD45 antibody (5 *μ*L), mouse anti-human CD294 antibody (5 *μ*L), and human lineage compound antibody (CD2, CD3, CD4, CD7, CD8, CD10, CD11b, CD14, CD19, CD20, CD56, and CD235a (20 *μ*L)) (BD Biosciences, America) at room temperature in the dark for 40 minutes and analyzed by FACSVerse flow cytometry (BD Biosciences, America).

### 2.4. Cell Culture and Stimulation

Human kidney proximal tubular epithelial cells (HK-2) were cultured in DMEM/F12 (HyClone, America) medium supplemented with 5% (vol/vol) heat-inactivated fetal bovine serum (Gibco/Life Technologies, America), 100 U/mL penicillin, and 100 *μ*g/mL streptomycin in an incubator with a controlled, humidified atmosphere containing 5% CO_2_. Cells were passaged with 0.25% trypsin when necessary. We divided the cells into 6 groups: (1) normal control (NC): DMEM/F12 culture medium alone (glucose concentration: 15.5625 mmol/L); (2) different concentrations of IL-4: 1, 10, or 100 ng/mL; (3) different concentrations of IL-13: 1, 10, or 100 ng/mL; (4) high glucose (HG): DMEM/F12 culture medium containing 30 mmol/L glucose; (5) IL-4+high glucose (HG+IL-4): 100 ng/mL IL-4+30 mmol/L glucose; and (6) IL-13+high glucose (HG+IL-13): 1 ng/mL IL-13+30 mmol/L glucose. Each test was independently repeated at least three times.

### 2.5. Enzyme-Linked Immunosorbent Assay (ELISA)

Plasma levels of IL-4, IL-5, and IL-13 (Beijing Cheng Lin Biological Technology Co. Ltd., China) were measured using an ELISA kit following the manufacturer's instructions. Culture supernatant levels of TGF-*β*1 and FN (Neobioscience Technology Company, China) were measured using an ELISA kit in accordance with the manufacturer's instructions. Concentrations were calculated according to the standard curve.

### 2.6. Quantitative Real-Time PCR (qPCR)

Expression levels of IL-4R*α*, IL-13R*α*1, TGF-*β*1, FN, and collagen1 genes were analyzed by qPCR. Briefly, total RNA was extracted from HK-2 cells using an RNA extraction kit (Beijing Tiangen Biotech, China), and total RNA was reversely transcribed into cDNA using random primers and a reverse transcriptase kit (TOYOBO, Japan) according to the manufacturer's instructions. The qPCR was performed using the iScript™ two-step qPCR kit with SYBR Green (Invitrogen). All gene primer sequences used are listed in Table 1. PCR was performed in triplicate on the real-time PCR detection system with the following cycling parameters: 95°C (2 min), 36 cycles of 95°C (5 s), and 60°C (10 s). The qPCR data were quantified using the 2^-ΔΔ^Ct method.

### 2.7. Statistical Analysis

All data are expressed as mean ± standard deviation (SD) for at least 3 independent experiments and analyzed by one-way analysis of variance (ANOVA), followed by the LSD post hoc test for multiple comparisons (SPSS17.0 statistical software).

## 3. Results

### 3.1. Baseline Characteristics

The baseline characteristics of the included subjects in the study are presented in Table 2. There were no significant differences in gender composition or age among the four groups (NC, DM, DKD1, and DKD2), but there were significant differences in body mass index (BMI), systolic blood pressure (SBP), fasting blood glucose (FBG), glycated hemoglobin (HbA1c), or white blood cell (WBC) number (*P* < 0.05).

### 3.2. The Proportion of ILC2s and Levels of IL-4, IL-5, and IL-13 Were Elevated in Patients with DKD

**Figure 1:**
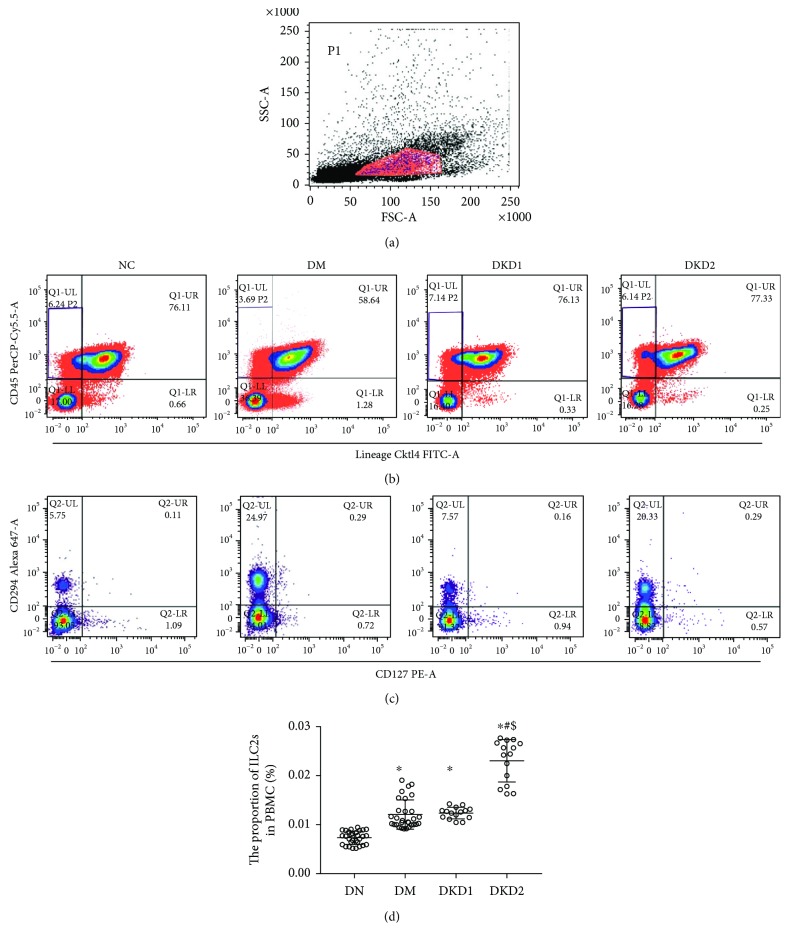
ILC2s were defined as Lin^−^CD45^+^CD127^+^CD294^+^ cells. (a) The amount of PBMC in the test sample. (b) The proportion of CD45^+^Lineage^−^ cells in the PBMC. (c) The proportion of CD294^+^CD127^+^ cells in the CD45^+^Lineage^−^ cells. (d) The proportion of ILC2s in peripheral blood PBMC in each group (NC *n* = 30, DM *n* = 30, DKD1 *n* = 15, and DKD2 *n* = 15). ^∗^*P* < 0.05 vs. NC group, ^#^*P* < 0.05 vs. DM group, ^$^*P* < 0.05 vs. DKD1 group.

The proportion of ILC2s in the peripheral blood PBMC was detected using flow cytometry. ILC2s were defined as Lin^−^CD45^+^CD127^+^CD294^+^ cells. Compared with the normal group, the proportion of ILC2s in the peripheral blood (PBMC) of the DM group and the DKD group was significantly increased (*P* < 0.05). Furthermore, the proportion of ILC2s in the DKD2 group was significantly higher than that in the DM group or DKD1 group (*P* < 0.05) (Figure 1).

**Figure 2:**
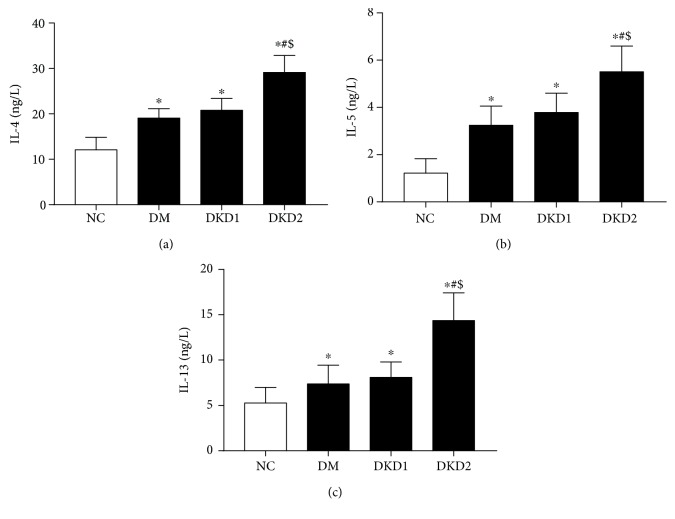
The plasma levels of IL-4, IL-5, and IL-13 (NC *n* = 30, DM *n* = 30, DKD1 *n* = 15, and DKD2 *n* = 15) ^∗^*P* < 0.05 vs. NC group, ^#^*P* < 0.05 vs. DM group, ^$^*P* < 0.05 vs. DKD1 group.

Compared with the NC group, the plasma levels of IL-4, IL-5, and IL-13 in the DM, DKD1, and DKD2 groups were significantly increased (*P* < 0.05). Compared with the DM and DKD1 groups, the levels of IL-4, IL-5, and IL-13 were increased in the DKD2 group (*P* < 0.05) (Figure 2). The trend of the change in cytokine levels was consistent with the proportion of ILC2s.

**Figure 3:**
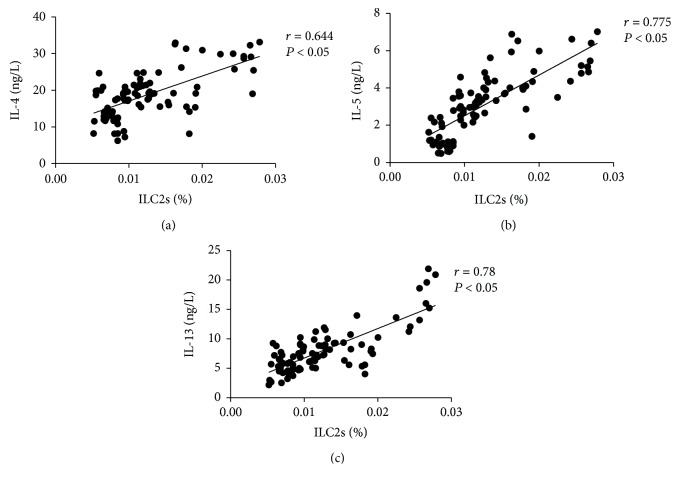
The correlation analysis between the proportion of ILC2s and the levels of Th2 cytokines (IL-4, IL-5, and IL-13).

**Figure 4:**
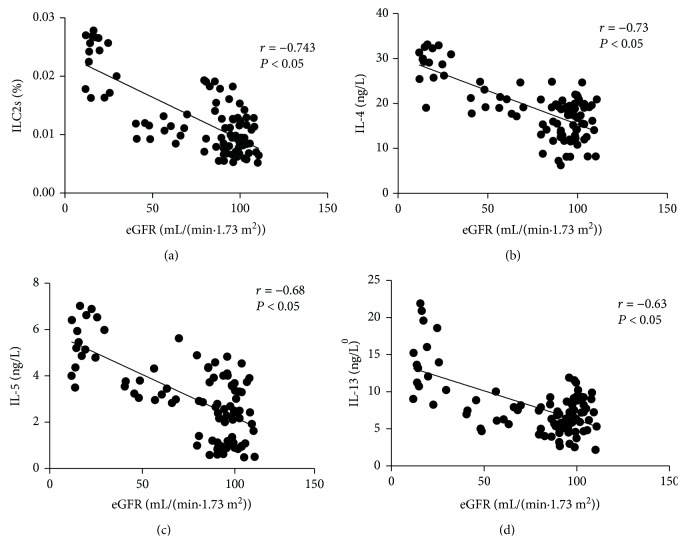
The correlation analysis between eGFR and the proportion of ILC2s and the levels of Th2 cytokines (IL-4, IL-5, and IL-13).

The proportion of ILC2s was positively correlated with the level of IL-4 (*r* = 0.644, *P* < 0.05), IL-5 (0.775, *P* < 0.05), and IL-13 (0.78, *P* < 0.05) (Figure 3). The level of eGFR was negatively correlated with the proportion of ILC2s (-0.743, *P* < 0.05), IL-4 (-0.73, *P* < 0.05), IL-5 (-0.68, *P* < 0.05), and IL-13 (-0.63, *P* < 0.05) (Figure 4).

### 3.3. IL-4 and IL-13 Can Upregulate the Expression of TGF-*β*1, FN, and Collagen1 in Cultured HK-2 Cells

**Figure 5:**
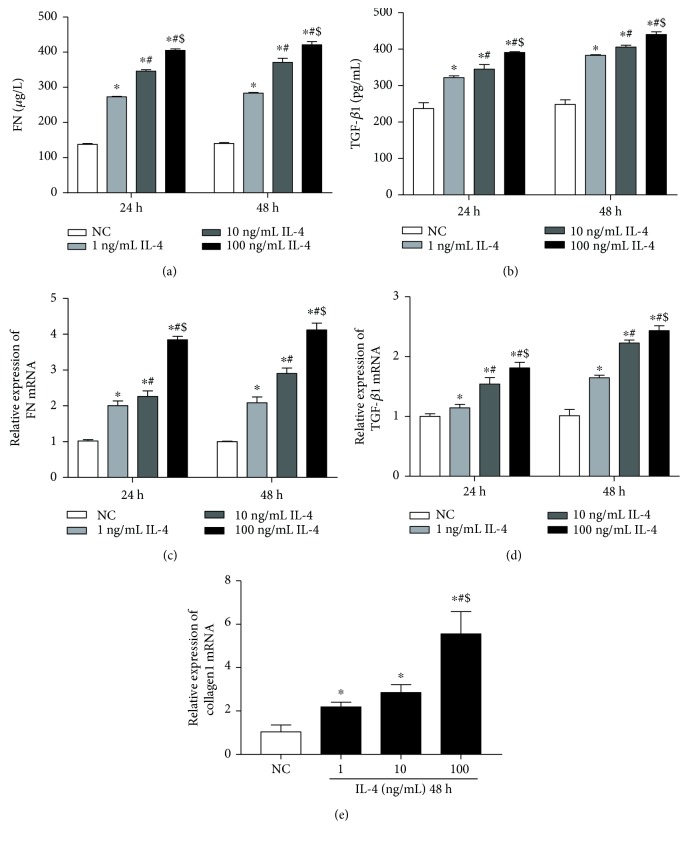
(a) The level of FN in cultural supernatant when cultured for 24 h and 48 h. (b) The level of TGF-*β*1 in cultural supernatant when cultured for 24 h and 48 h. (c) The relative expression of FN mRNA when cultured for 24 h and 48 h. (d) The relative expression of TGF-*β*1 mRNA when cultured for 24 h and 48 h. (e) The relative expression of collagen1 mRNA when cultured for 48 h. NC group means HK2 cells were incubated with DMEM/F12 alone (each group *n* = 3). ^∗^*P* < 0.05 vs. NC group, ^#^*P* < 0.05 vs. 1 ng/mL IL-4 group, ^$^*P* < 0.05 vs. 10 ng/mL IL-4 group.

Compared with the NC subjects, the levels of TGF-*β*1 and FN in cell supernatants and the relative expression of the TGF-*β*1 mRNA, FN mRNA, and collagen1 mRNA increased significantly in all groups receiving IL-4 stimulation for 24 h or 48 h (Figure 5). The expression levels of these genes were the highest in the 100 ng/mL IL-4 group (*P* < 0.05). The FN levels in cell culture supernatants and FN mRNA were slightly higher in cells treated with IL-4 for 48 h than those in the treated cells for 24 h (*P* < 0.05) (Figures 5(a) and 5(b)). Similarly, both the level of TGF-*β*1 in cell culture supernatant and TGF-*β*1 mRNA were slightly higher after 48 h treatment with IL-4 than those for 24 h treatment (Figures 5(c) and 5(d)). IL-4 stimulation can promote the expression of TGF-*β*1 and FN in HK-2 cells in a concentration-dependent and time-dependent manner.

**Figure 6:**
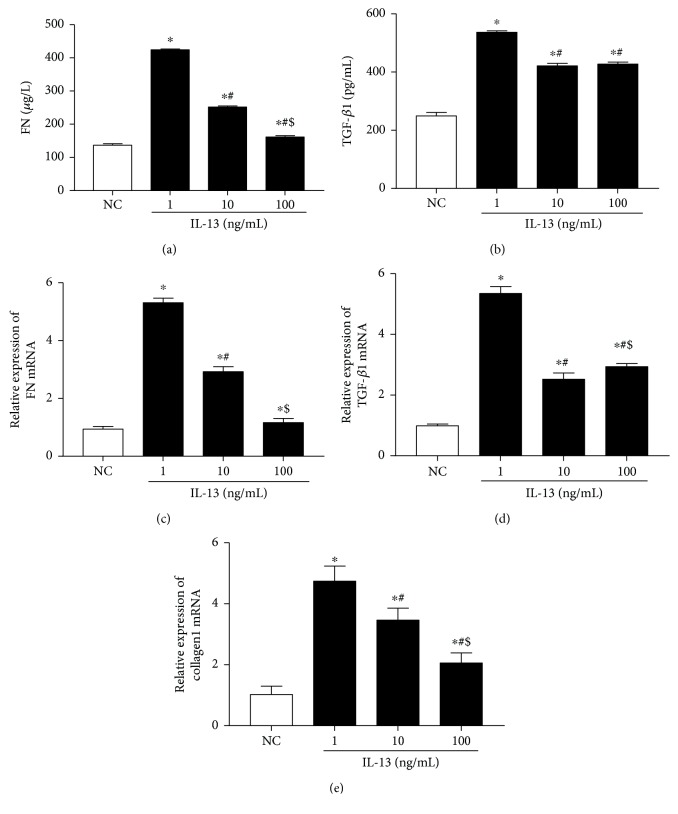
(a, b) The levels of FN and TGF-*β*1 in cultural supernatant. (c–e) The relative expression of FN mRNA, TGF-*β*1 mRNA, and collagen1 mRNA. Cells were cultured for 24 h. NC group means HK2 cells were incubated with DMEM/F12 alone (each group *n* = 3). ^∗^*P* < 0.05 vs. NC group, ^#^*P* < 0.05 vs. 1 ng/mL IL-13 group, ^$^*P* < 0.05 vs. 10 ng/mL IL-13 group.

The relative expression of FN mRNA in HK-2 cells treated with 100 ng/mL IL-13 was higher than that in the untreated cells. However, the difference was not significant (*P* > 0.05) (Figure 6(c)). The relative expression of TGF-*β*1 mRNA, FN mRNA, and collagen1 mRNA and the levels of FN and TGF-*β*1 in culture supernatant in the 1 and 10 ng/mL IL-13-treated cells were all significantly increased (*P* < 0.05) (Figure 6). The relative expression of TGF-*β*1 mRNA, FN mRNA, and collagen 1 mRNA in HK-2 cells treated with 1 ng/mL IL-13 was the highest among all groups (*P* < 0.05) (Figure 6). It is suggested that IL-13 stimulation can promote the expression of TGF-*β*1, FN, and collagen1 in HK-2 in vitro.

### 3.4. High Glucose with or without IL-4 and IL-13 Cannot Promote the Expression of IL-4R*α* and IL-13R*α*1 in Cultured HK-2 Cells

**Figure 7:**
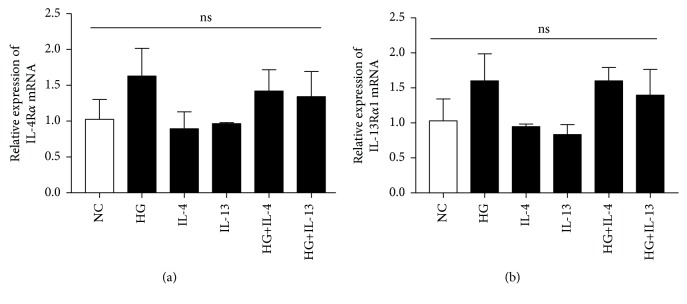
The relative expression of IL-4R*α* mRNA and IL-13R*α*1 mRNA. Cells were cultured for 48 h. NC means HK2 cells were incubated with DMEM/F12 alone. HG means HK2 cells were incubated with DMEM/F12 culture medium containing 30 mmol/L glucose. The concentration of IL-4 was 100 ng/mL. The concentration of IL-13 was 1 ng/mL (each group *n* = 3). ns: *P* > 0.05 between all groups.

We analyzed the expression of IL-4R*α* and IL-13R*α*1 in high glucose-treated HK-2 cells which were cocultured with or without IL-4 or IL-13 for 48 hours. Compared with the NC group, the expressions of IL-4R*α* mRNA and IL-13R*α*1 mRNA were upregulated in HK2 cells when stimulated with high glucose alone or high glucose combined with IL-4 or IL-13, but the differences were not statistically significant (*P* > 0.05) (Figure 7).

### 3.5. IL-4 and IL-13 Can Synergize with High Glucose to Promote the Expression of TGF-*β*1, FN, and Collagen1 in Cultured HK-2 Cells

**Figure 8:**
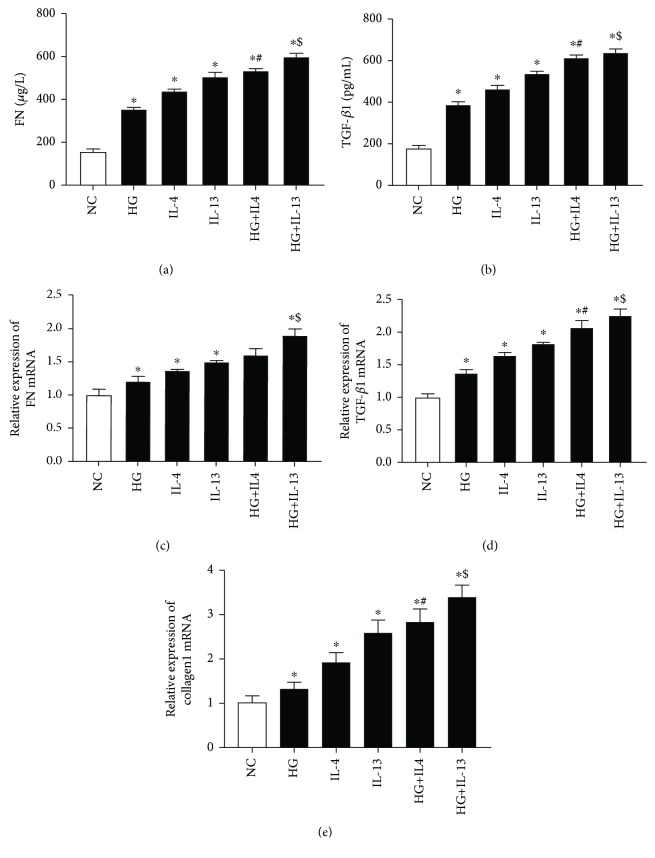
(a, b) The levels of FN and TGF-*β*1 in cultural supernatant. (c–e) The relative expression of FN mRNA, TGF-*β*1 mRNA, and collagen1 mRNA. Cells were cultured for 48 h. NC means HK2 cells were incubated with DMEM/F12 alone. HG means HK2 cells were incubated with DMEM/F12 culture medium containing 30 mmol/L glucose. The concentration of IL-4 was 100 ng/mL. The concentration of IL-13 was 1 ng/mL (each group *n* = 3). ^∗^*P* < 0.05 vs. NC group, ^#^*P* < 0.05 vs. IL-4 group, ^$^*P* < 0.05 vs. IL-13 group.

We analyzed the effects of IL-4 or IL-13 on the expression of TGF-*β*1, FN, and collagen1 levels in the HK-2 cells stimulated by high glucose for 48 hours (Figure 8). Compared with the NC group, the relative expression of TGF-*β*1 mRNA, FN mRNA, and collagen1 mRNA and the levels of FN and TGF-*β*1 in culture supernatant were significantly increased in the HG group, IL-4 group, IL-13 group, HG+IL-4 group, and HG+IL-13 group (*P* < 0.05). Compared with the IL-4 group, the expression levels of the TGF-*β*1, FN, and collagen1 increased significantly in the HG+IL-4 group (*P* < 0.05). The expression levels of TGF-*β*1, FN, and collagen1 in the HG+IL-13 group were significantly higher than those in the IL-13 group (*P* < 0.05). These results suggest that IL-4 and IL-13 can synergize with high glucose to promote the expression of TGF-*β*1, FN, and collagen1 in cultured HK-2 cells.

## 4. Discussion

Emerging evidences have shown that ILC2s mediate multiple types of tissue fibrosis such as intestinal fibrosis, hepatic fibrosis, and pulmonary fibrosis by secreting Th2 cytokines and regulating type 2 immunity [10, 15]. Renal interstitial inflammation and fibrosis are basic features in the progression of DKD. Previous studies have shown that DKD is associated with immunity [13, 25, 26]. In our study, compared with healthy volunteers, the proportion of ILC2s and the levels of IL-4, IL-5, and IL-13 were increased in DM and DKD, and this is consistent with previous researches [25]. Interestingly, the proportion of ILC2s was significantly increased with the severity of DKD. At the same time, there was a positive correlation between the proportion of ILC2s and the levels of Th2 cytokines (IL-4, IL-5, and IL-13). But ILC2s, IL-4, IL5, and IL-13 were negatively correlated with eGFR. Studies in the relationship between ILC2s and glycolipid metabolism were not yet mature. It was reported [27] that ILC2s promoted weight loss and improved insulin resistance in obese mice via the induction and maintenance of eosinophils and alternatively activated macrophages. Another study [25] found that the expression of ILC2-related factors RoR*α*, T1/ST2, IL-5, and IL-13 was elevated in patients with DKD and was positively correlated with body weight, fasting blood glucose, and blood lipids. Thus, in the early stage of DKD, ILC2s may be activated to participate the process of chronic kidney injury that is caused by persistent hyperglycemia. And ILC2s may also play a protective role by regulating glycolipid metabolism. In the progress of DKD, ILC2s were overactivated and a large amount of Th2 cytokines were secreted, which would lead to tissue fibrosis [10].

TGF-*β*1 plays a key role in mesangial cell proliferation, extracellular matrix (ECM) deposition, and epithelial-mesenchymal transition (EMT) through the Smads and MAPKS signaling pathways, ultimately leading to renal fibrosis [28, 29]. In our study, we found that the expression and release of TGF-*β*1, FN, and collagen1 were significantly upregulated in HK-2 cells induced by IL-4 or IL-13. In the high-glucose group, the expression of IL-4R*α* mRNA and IL-13R*α*1 mRNA in HK-2 cells was not upregulated, but the expression of TGF-*β*1, FN, and collagen1 was increased. Therefore, other fibrotic mediators may be secreted by HK2 cells under the stimulation with high glucose. Combined with high-glucose stimulation, IL-4 or IL-13 significantly upregulated the expression of TGF-*β*1, FN, and collagen1. These results suggested that IL-4 and IL-13 may trigger EMT in HK2 cells partly via TGF-*β*1 signal pathway and promote the progression of renal fibrosis in DKD.

The mechanism by which ILC2s regulate renal fibrosis in DKD is complex. Our study found that the secretion of the Th2 cytokines (IL-4, IL-13) and the upregulation of TGF-*β*1 were the key factors leading to renal fibrosis in DKD. ILC2s, activated by IL-25, IL-33, and TSLP, are involved in the development and progression of DKD by secreting IL-4 and IL-13 directly. IL-4 can promote Th2 cell proliferation by activating STAT6, and the Th2 cells can secrete more IL-4 [12]. Moreover, ILC2s can promote the differentiation of naive CD4^+^ T cells to a Th2 phenotype and inhibit Th1 differentiation in a contact-dependent manner [30]. Type 2 immunity is helpful in maintaining the homeostasis of tissue after bodily injury. However, when it becomes overexuberant or dysregulated, it can also contribute to the development of pathological fibrosis in tissue via secreting of Th2 cytokines [10, 11]. We found that IL-4 and IL-13 can promote HK2 cells to secrete TGF-*β*1. These Th2 cytokines can also promote the eosinophil and tissue repair macrophage to secrete TGF-*β*1 [31]. The ILC2-related Th2 cytokines (IL-4, IL-13) promote the expression of TGF-*β*1, leading to renal fibrosis via Smads and MAPKS signaling pathways. Besides, it has been reported that in IL-4 and IL-13-treated HK-2 cells, the JAK-STAT6 signaling pathway was sufficiently activated to promote EMT, mesangial cell proliferation, ECM deposition, and foot cell damage, eventually leading to renal fibrosis [32]. IL-13, another important cytokine produced by ILC2s, induces pathological tissue remodeling as well as fibrosis through IL-4R-dependent signal [33].

In conclusion, our present study found that the proportion of ILC2s and the levels of Th2 cytokines were significantly increased with the severity of DKD. Moreover, IL-4 and IL-13 upregulated the expression of TGF-*β*1, FN, and collagen1. These data suggest that ILC2s participate in renal fibrosis in DKD partly via TGF-*β*1 signal pathway. However, our research still has some shortcomings. First, the clinical sample size is small. Additionally, the mechanism by which ILC2s and the Th2 cytokines are involved in diabetic renal fibrosis requires further investigation. High selective agonists and antagonists for ILC2-related cytokine receptors as well as ILC2 knockout or overexpression mice will be required for future studies to elucidate the molecular mechanisms involved in ILC2-mediated renal fibrosis in DKD.

## Figures and Tables

**Table 1:** Gene primer sequences were used for qPCR analysis.

Gene	Forward primers (5′–3′)	Reverse primers (3′–5′)
Human FN	5′-TGTTATGGAGGAAGCCGAGGTT-3′	5′-GCAGCGGTTTGCGATGGT-3′
Human TGF-*β*1	5′-CCCACAACGAAATCCATGAC-3′	5′-gCTGAGGTATCGCCAGGAAT-3′
Human GAPDH	5′-CCACTCCTCCACCTTTG-3′	5′-CACCACCCTGTTGCTGT-3′
Human collagen1a1	5′-CCAAATATGTCTCCCCAGAA-3′	5′-TCAAAAACGAAGGGGAGATG-3′
Human IL-4R*α*	5′-CAAGCTCTTGCCCTGTTTTC-3′	5′-TGCACAGAAGCTCCCTTTTT-3′
Human IL-13R*α*1	5′-AGATGGCCATGAAGAGGATG-3′	5′-CCCAAGACCTAGGGATCACA-3′

**Table 2:** Characteristics of the normal control subjects (NC) or patients with type 2 diabetes (DM), early diabetic kidney disease (DKD1), and late diabetic kidney disease (DKD2) at baseline.

Items	NC	DM	DKD1	DKD2	*F*	*P*
Gender (male/female)	16/14	16/14	9/6	8/7		
Age (year)	61.03 ± 7.95	60.19 ± 9.71	64.70 ± 9.95	62.20 ± 7.85	1.771	0.156
BMI (kg/m^2^)	23.55 ± 2.59	24.87 ± 3.83	26.14 ± 4.00	23.42 ± 6.29	3.020	0.032
SBP (mmHg)	124 ± 17	128 ± 15	141 ± 14	144 ± 23	9.248	<0.01
DBP (mmHg)	75 ± 12	73 ± 17	77 ± 10	87 ± 14	1.609	0.190
FBG (mmol/L)	4.8 ± 0.5	8.8 ± 2.1	9.1 ± 2.5	10 ± 2.6	41.083	<0.01
HbA1c (%)	5.4 ± 0.4	8.8 ± 2.0	8.5 ± 1.9	7.5 ± 1.2	34.566	<0.01
WBC (10^9^/L)	4.83 ± 0.79	6.46 ± 1.57	7.3 ± 1.56	6.81 ± 1.5	19.205	<0.01
Neu (10^9^/L)	3.03 ± 0.71	4.07 ± 1.25	5.00 ± 1.51	4.74 ± 1.26	16.688	<0.01
Lym (10^9^/L)	1.39 ± 0.25	1.78 ± 0.58	1.61 ± 0.52	1.39 ± 0.52	5.594	<0.01
TG (mmol/L)	1.19 ± 0.35	2.06 ± 1.21	2.41 ± 1.49	2.11 ± 1.32	7.097	<0.01
TC (mmol/L)	4.86 ± 0.74	4.4 ± 1.26	4.97 ± 1.50	4.33 ± 1.06	2.362	0.074
HDL-C (mmol/L)	1.51 ± 0.49	1.21 ± 0.36	1.04 ± 0.26	1.11 ± 0.27	10.173	<0.01
LDL-C (mmol/L)	2.80 ± 0.63	2.94 ± 0.63	3.24 ± 0.78	2.98 ± 0.68	2.553	0.058
Cr (*μ*mol/L)	61.96 ± 9.29	62.65 ± 20.10	101.19 ± 36.50	303.77 ± 79.75	240.383	<0.01
UA (*μ*mol/L)	297.21 ± 69.73	307.38 ± 87.00	368.49 ± 84.69	421.12 ± 90.60	11.670	<0.01
UAER (mmol/L)	5.38 ± 1.08	5.79 ± 2.06	9.09 ± 5.41	14.37 ± 4.98	32.558	<0.01
eGFR (mL/min·1.73 m^2^)	95.24 ± 14.13	97.91 ± 18.26	61.30 ± 20.49	18.11 ± 7.40	108.749	<0.01

BMI: body mass index, SBP: systolic pressure, DBP: diastolic pressure, FBG: fasting blood glucose, HbA1c: glycosylated hemoglobin A1c, WBC: white blood cell, Neu: neutrophil, Lym: lymphocyte, TG: triglyceride, TC: cholesterol, HDL-C: high-density lipoprotein cholesterol, LDL-C: low-density lipoprotein cholesterol, Cr: creatinine, UA: uric acid, eGFR: estimated glomerular filtration rate. The *F* and *P* represent the results of comparison between groups.

## Data Availability

The data used to support the findings of this study are available from the corresponding author upon request.
